# Early Use of Phenol Neurolysis Likely Reduces the Total Amount of Botulinum Toxin in Management of Post-Stroke Spasticity

**DOI:** 10.3389/fresc.2021.729178

**Published:** 2021-09-16

**Authors:** Sheng Li, Jean Woo, Manuel F. Mas

**Affiliations:** ^1^Department of Physical Medicine and Rehabilitation, The NeuroRecovery Research Center, McGovern Medical School, TIRR Memorial Hermann, University of Texas Health Science Center, Houston, TX, United States; ^2^H. Ben Taub Department of Physical Medicine and Rehabilitation, Baylor College of Medicine, Houston, TX, United States; ^3^Department of Physical Medicine, Rehabilitation and Sports Medicine, School of Medicine, University of Puerto Rico, San Juan, PR, United States

**Keywords:** spasticity, stroke, botulinum toxin, motor recovery, rehabilitation, phenol

## Abstract

The main objective was to examine practice patterns of phenol neurolysis for post-stroke spasticity management in the early stage. We performed a chart review of patients who were admitted for inpatient rehabilitation within 6 months after first-ever stroke and received phenol neurolysis within 15 months post-stroke. Out of 2,367 stroke admissions from January 2014 and December 2018, 68 patients met the criteria. 52.9% of these patients received phenol neurolysis within 12 weeks, i.e., early stage. The earliest phenol neurolysis procedure was at 19 days after stroke. On average, patients received first phenol injections at 16.3 weeks after stroke with an average dose of 7.3 ml. Most commonly injected nerves were tibial nerve motor branches (41/68), sciatic nerve motor branches (37/68), lateral pectoral nerve (16/68), medial pectoral nerve (15/68), obturator nerve (15/68) and musculocutaneous nerve (15/68). Among 68 patients, 24 received phenol only; 17 received phenol neurolysis first followed by botulinum toxin (BoNT) injections; 19 received BoNT injections first followed by phenol neurolysis; 8 received both phenol and BoNT injections at the same time. The interval from stroke to first procedure was similar between the Phenol-First group (13.3 weeks) and the BoNT-First group (12.6 weeks). The total amount of BoNT was significantly lower in the Phenol-First group (361.3 units) than in the BoNT-First group (515.8 units) (*p* = 0.005). The total amount of phenol was not statistically different between the Phenol-First group (5.9 ml) and the BoNT-First group (8.3 ml). The interval between the first procedure and its subsequent procedure was not statistically different between the Phenol-First group (18.3 weeks) and the BoNT-First group (10.7 weeks). These long intervals suggest that the subsequent injection (type and dose) was not planned during the first procedure. The general patterns of target areas were similar between BoNT injections and phenol neurolysis, except that phenol neurolysis rarely targeted the upper extremity distal muscles. No side effects after phenol or BoNT injections in the early stage after stroke were observed in the chart review. In summary, phenol neurolysis was started as early as 19 days after stroke. On average, patients received first phenol about 4 months after stroke with an average of 7.3 ml of phenol. Early use of phenol neurolysis likely decreases the total amount of BoNT for management of post-stroke spasticity without increased side effects.

## Introduction

Spasticity and muscle weakness on the affected side (i.e., hemiparesis) are two hallmark motor impairments after stroke. Recent advances have shown that these two motor impairments are mediated by distinctly different pathophysiological mechanisms (1–4). Hemiparesis, as a result of damage of motor cortex and/or its descending corticospinal tracts, occurs immediately after stroke. In contrast, spasticity develops gradually as a phenomenon of maladaptive neuroplasticity. It emerges as early as 2 weeks (5), and progressively worsens over time (6). The prevalence of spasticity is 19% by 3 months (7), and 43.2% by a year post stroke (8). These two motor impairments usually interact with each other and amplify the problems in a vicious cycle (9, 10), thus causing consequences in motor rehabilitation for stroke survivors. However, early intervention of spasticity management is likely to interrupt this cycle. In a recent study (5), stroke survivors received botulinum toxin (BoNT) injections early at spasticity emergence with a mean time of 18 days post stroke. As compared to the control group, the intervention group demonstrated significantly greater reduction in spasticity development, higher passive range of motion and less contracture formation. Given known side effects of muscle weakness from BoNT injection, the results of similar motor recovery between two groups are encouraging. In a prospective, longitudinal study, Picelli et al. (11) reported that early BoNT injections (<3 months) had better and longer lasting effects of spasticity reduction than late BoNT injections (>3 months). Similarly, in our retrospective study (12), we found that stroke survivors who received BoNT injections at an earlier time had much longer lasting effects than those who received injections at a late date. Taken together, these studies have supported the benefits of early spasticity intervention after stroke (5, 13–19).

Phenol neurolysis is another effective intervention to manage spasticity. As compared to BoNT injections, phenol neurolysis is used less frequently and less studied (20). Advances in guidance techniques, such as ultrasound imaging and intramuscular electrical stimulation, make precise localization of nerves and nerve branches possible. Side effects, especially worrisome dysesthesia, are greatly minimized. In our recent retrospective studies (21), side effects were low in general, and dysesthesia was found only in 2 out 293 of procedures (0.7%). With combined ultrasound and electrical stimulation guidance, we did not find any documented report of dysesthesia after phenol neurolysis for the distal upper extremity muscles (22). Phenol neurolysis for nerve block have shown similar or better efficacy in spasticity reduction as compared to BoNT injection (23, 24). When used together, phenol neurolysis is often used for proximal muscles, while BoNT is for distal muscles (21, 25, 26). In these studies, phenol neurolysis is mostly used in the chronic stage. Given the immediate effect of phenol neurolysis (27), it could be a useful intervention for spasticity management in the early stage during inpatient rehabilitation.

The main objective was to examine practice patterns of phenol neurolysis for post-stroke spasticity management in the early stage in this a retrospective chart review study. Specially, we were interested in a few real-world practice questions, such as, how early was phenol neurolysis used after stroke? what were target nerves and nerve branches, and doses? how did early phenol neurolysis affect subsequent BoNT injections? and any side effects associated with early phenol neurolysis?

## Methods

### Subjects

This retrospective study was approved by the Committee for the Protection of Human Subjects of UTHealth (HSC-MS-18-0198). The inclusion criteria included that patients (1) suffered first-ever stroke; (2) were admitted within 6 months after stroke to our acute inpatient rehabilitation hospital from January 2014 and December 2018; and (3) received first phenol injections for spasticity management within 15 months of stroke. Post-stroke spasticity at this institution is treated when it becomes problematic, such as when it limits therapy participation or causes pain. The decision of interventional management (phenol and/or BoNT) is determined by consensus of clinical judgement of physiatrists and members of the rehabilitation team (physical therapy, occupational therapy, and nursing). The physiatrists decide which intervention, or combination of interventions, target muscles/nerves, and doses. Exclusion criteria were presence of premorbid muscle hypertonicity, prior medical conditions of intracranial or spinal cord origin, presence of intrathecal baclofen pump prior to phenol/BoNT injections, and chronic stroke older than 6 months at the time of admission.

### Data Collection and Analysis

Data collected include patient demographics, onset date and etiology of stroke, and dates, doses and nerves targeted of phenol injections. If the patient was also injected with BoNT for spasticity management before or after phenol injection, procedure data were collected as well (date of procedure, muscles injected, dose distribution and total dose). Different types of BoNT (ona-, inco-, and abo-BoNT) were used at this institution. However, we did not find any switch between BoNTs in the consecutive injections for any particular patient. In general practice, the absolute number of units was higher in abo-BoNT than in ona- or inco-BoNT to achieve similar clinical outcomes. Although there is no definitive evidence for converting units among BoNTs, we adopted the conversion rate we recently used (12). The total dose of abo-BoNT was converted to an ona-BoNT equivalent dose in a 3:1 ratio, while a 1:1 ratio was used between ona-BoNT and inco-BoNT. Patients were subdivided into four groups: (a) those who only received phenol injections (Phenol-Only), (b) those who received phenol injection first and then BoNT injection on a later date (Phenol-First); (c) those who received BoNT injection first and a phenol injection on a later date (BoNT-First) and (d) those who received BoNT and phenol injections during their first injections (Both-First). In our institution, 6% aqueous phenol was used for neurolysis. For botulinum toxins, different physiatrists may use different dilutions (1:1 or 1:2 [100 units in 2 ml normal saline]). All patients received standard 3-h inpatient therapy before and after phenol or BoNT injections in this chart review study.

Descriptive statistics were used for demographics and characteristics of first injections. Variables included interval between procedures, total dose of phenol and total dose of BoNT. Groups B (Phenol-First) and C (BoNT-First) were compared to examine the effects on phenol neurolysis on the subsequent injections. Repeated measure two-way ANOVA tests were performed. Factors included INTERVENTION (2 levels, Phenol_First vs. BoNT_First), and AREA (5 levels, neck, upper proximal, upper distal, lower proximal, and lower distal areas). Tukey's *Post-hoc* tests were used to detect the differences. The level of significance was set at *p* < 0.05.

## Results

Out of 2,367 stroke admissions from January 2014 and December 2018, 68 patients met the criteria. Among 68 patients, 24 received phenol injections only; 17 received phenol injections first followed by BoNT injections; 19 received BoNT injections first followed by phenol neurolysis (BoNT-first group); Eight received both phenol and BoNT injections at the same time (Table 1).

**Table 1:** Demographics of all phenol-injected subjects.

	**Phenol-first**	**BoNT-first**	**Both-first**	**Phenol-only**
	**(*N* = 17)**	**(*N* = 19)**	**(*N* = 8)**	**(*N* = 24)**
Average age at stroke	57.9 ± 16.8	51.5 ± 13.3	54.9 ± 11.7	58.0 ± 13.0
Sex; *N* (%)				
Male	10 (58.8%)	10 (52.6%)	6 (75%)	11 (45.8%)
Female	7 (41.2 %)	9 (47.4%)	2 (25%)	13 (54.2%)
Types of stroke; *N* (%)				
Ischemic	10 (58.8%)	8 (42.1%)	4 (50%)	13 (42.2%)
Non-ischemic	7 (41.2%)	11 (57.9%)	4 (50%)	11 (45.8%)

*N = 68*.

### Phenol Injection Characteristics

**Figure 1:**
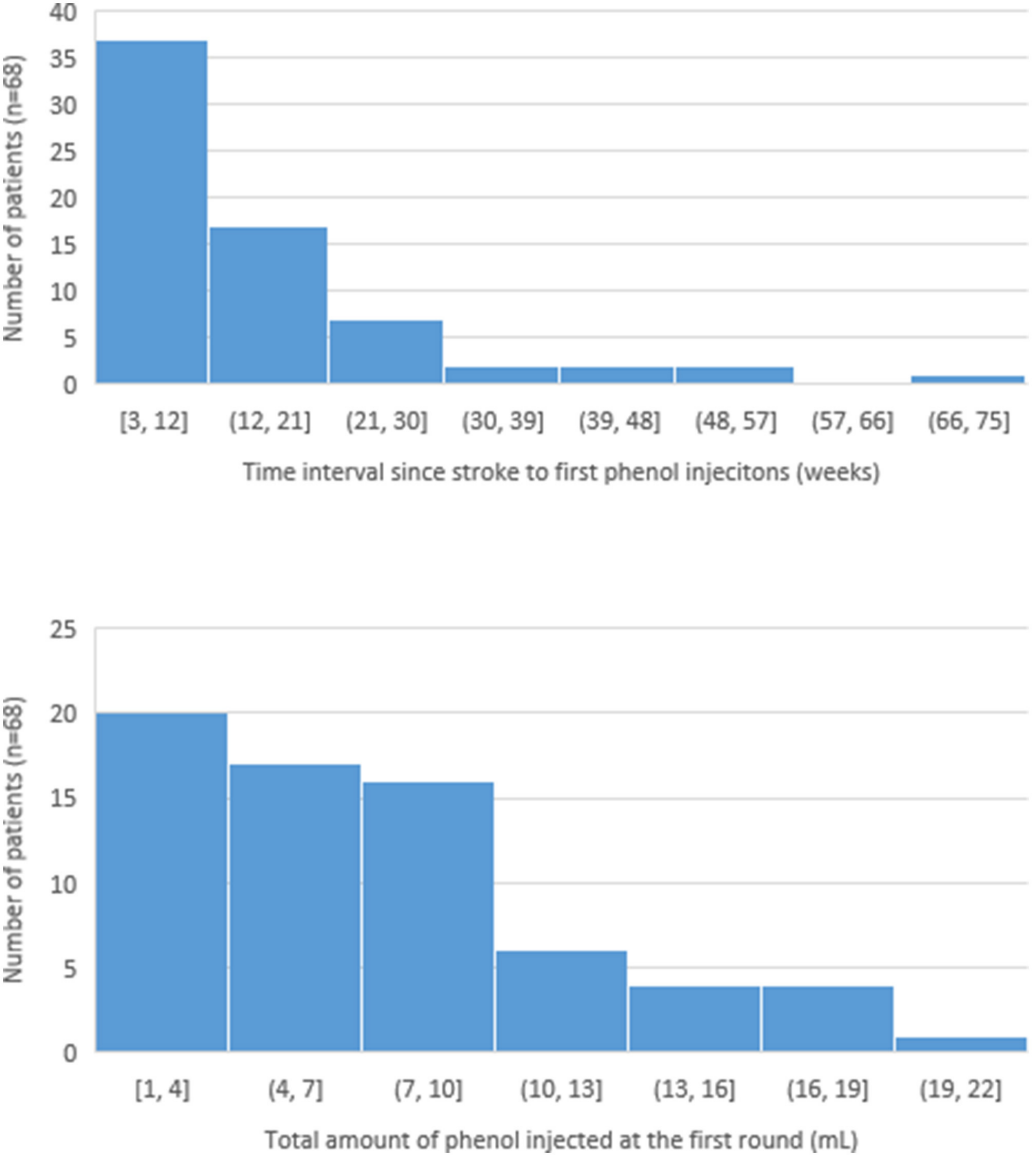
Distribution of the time since stroke to first phenol injection and the total amount of phenol injected at the first round.

Among 68 patients, the average duration between the stroke and the first phenol injection was 15.0 weeks. The earliest phenol injection was performed at 2.7 weeks (19 days) after stroke onset. About 52.9% of patients (36 out of 68) received phenol neurolysis within 12 weeks after stroke (Figure 1). The dose of phenol per procedure was variable, ranging from 1ml to 20 ml. Average dose injected at the first round of phenol was 7.4 ml. However, 29.4% of patients received <4.0 ml; 77.9% patients received <10.0 ml. The distribution of the average interval between stroke and the first injection as well as the total amount of phenol injected at the first round are depicted in Figure 1.

Phenol was commonly injected to the proximal muscles than the distal muscles of the extremities (Table 2). The most commonly injected nerves or nerve branches were tibial (60.3%) and sciatic (54.4%) nerves/branches. The nerves innervating distal muscles of the limbs were rarely injected. On average, the dose was higher for the nerves in the lower extremity than in the upper extremity.

**Table 2:** Number of patients that received injection to different nerves and nerve branches and the average dose of phenol injected to each nerve/branch among 68 patients.

**Targeted nerves during the first round**		**Number and % of patients who received phenol injection to each nerve**	**Average dose (ml) ±SD**
Medial pectoral	Upper proximal	15 / 68, 22.1%	1.9 (±1.3)
Lateral pectoral		16 / 68, 23.5%	1.7 (±0.9)
Thoracodorsal		3 / 68, 4.4%	2.3
Musculocutaneous		15 / 68, 22.1%	2.5 (±1.2)
Median (FCR)	Upper distal	2 / 68, 2.9%	0.8
Ulnar (FCU)		1 / 68, 1.5%	0.5
Radial (brachioradialis)		1 / 68, 1.5%	1.0
Femoral	Lower proximal	5 / 68, 7.4%	5.0
Sciatic		37 / 68, 54.4%	4.5 (±2.9)
Obturator		15 / 68, 22.1%	3.9 (±2.1)
Peroneal	Lower distal	1 / 68, 1.5%	5.0
Tibial		41 / 68, 60.3%	4.0 (±2.6)

### Comparisons Between Phenol-First vs. BoNT-First Injections

**Figure 2:**
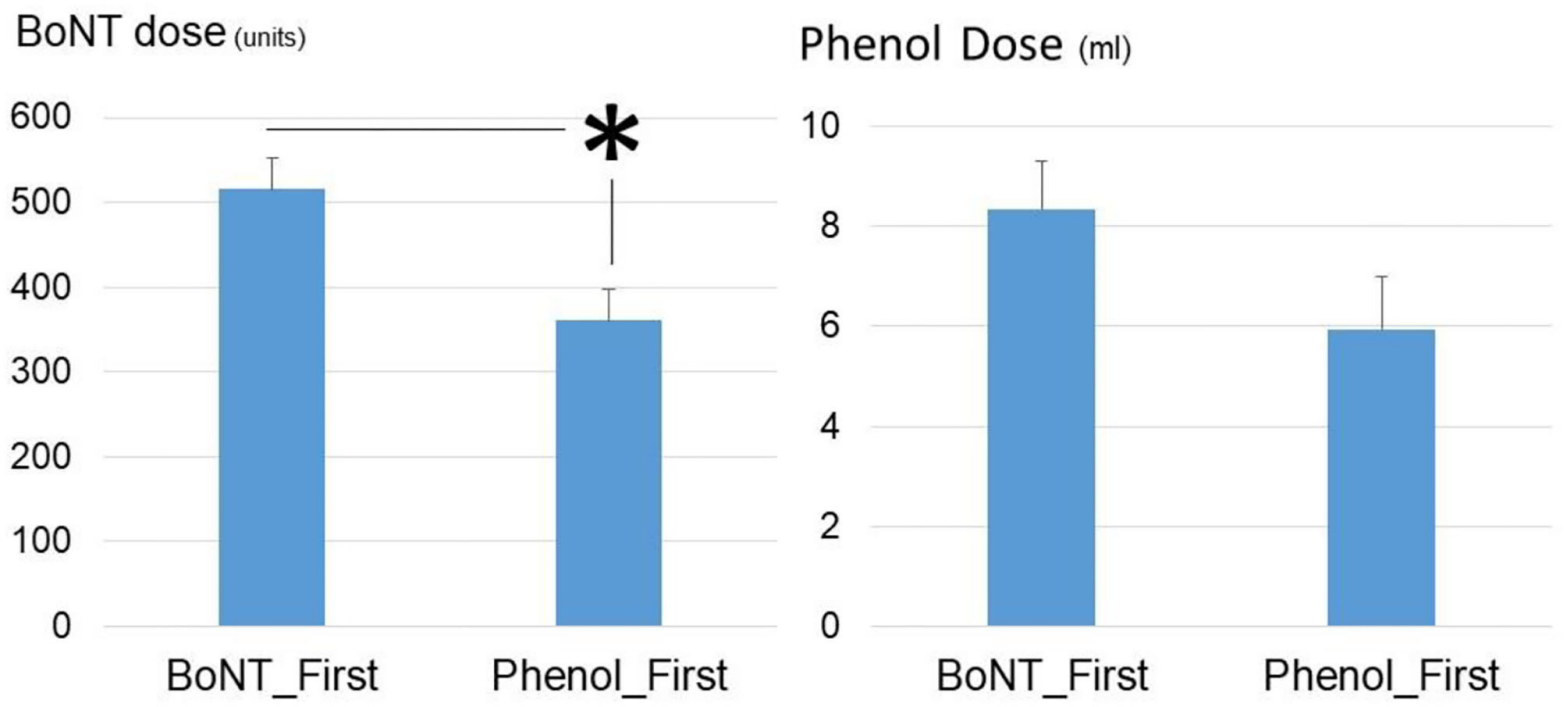
Comparisons of phenol dose and BoNT dose between the Phenol_First group and the BoNT_First group. Asterisk: statistically significant difference. Standard errors were shown.

In order to assess the effect of first injection on the subsequent injections, Phenol-First (*N* = 17) and BoNT-First (*N* = 19) groups were compared (Figure 2). The interval from stroke to first procedure was similar between the Phenol-First group (13.3 ± 2.1 weeks) and the BoNT-First group (12.6 ± 1.8 weeks) (*p* = 0.79). The interval between the first procedure and its subsequent procedure was longer in the Phenol-First group (18.3 ± 3.6 weeks) than in the BoNT-First group (10.7 ± 3.9 weeks), but the difference was not statistically significant (*p* = 0.15). The order of intervention (Phenol vs. BoNT) did affect the total dose in the subsequent injection. The total amount of BoNT was significantly lower in the Phenol-First group (361.3 ± 35.8 units) than in the BoNT-First group (515.8 ± 36.9 units) (*p* = 0.005). The total amount of phenol was also smaller in the Phenol-First group (5.9 ± 1.1 ml) than in the BoNT-First group (8.3 ± 0.9 ml), but the difference was not statistically significant (*p* = 0.097).

**Figure 3:**
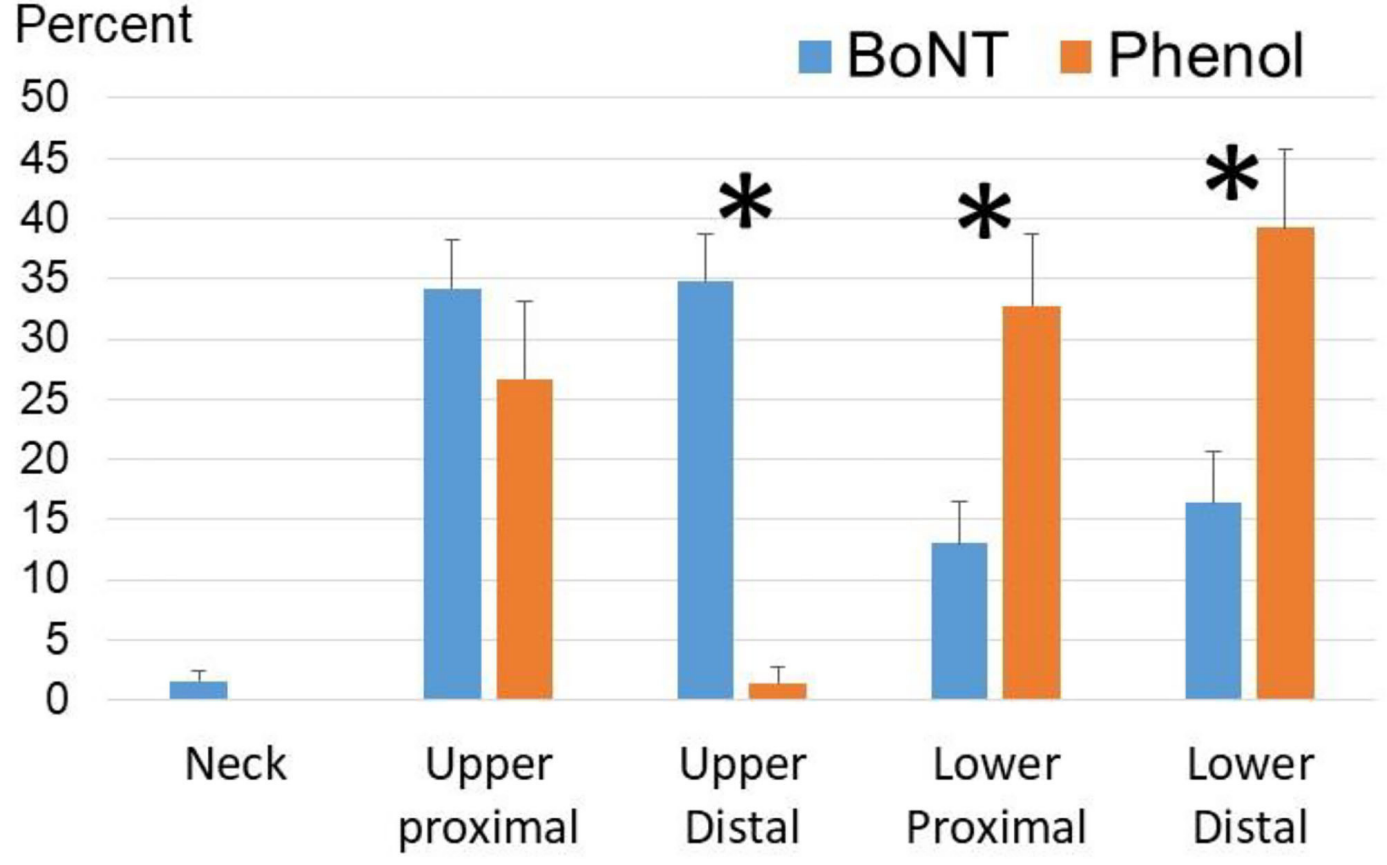
Frequently targeted areas in phenol and BoNT injections among the Phenol-First and BoNT-First groups combined (*N* = 36). For BoNT injections, the upper proximal area includes shoulder and elbow muscles; the upper distal area includes wrist and finger muscles; the lower proximal area includes hip and knee muscles; the lower distal area includes ankle and foot muscles. For phenol injections, the upper proximal area includes medial and lateral pectoral nerves, thoracodorsal nerve, and musculocutaneous nerve; the upper distal area includes radial, median and ulnar nerves and their branches; the lower proximal area includes femoral, obturator and sciatic nerves and their motor branches; the lower distal area includes peroneal and tibial nerve branches. Asterisks: statistical significance. Standard errors were shown.

To better compare distribution patterns, injections were grouped into these five areas: neck, upper proximal, upper distal, lower proximal, and lower distal (see Figure 3). Percent for each area was calculated by the number of injections in this area (muscles or nerves) out of the total number of injections for each procedure. According to repeat two-way ANOVAs, there was no statistically significant difference in the distribution pattern to different areas between to interventions (Phenol vs. BoNT). However, main effects of AREA [*F*_(4,32)_ = 427.0, *p* < 0.001] and AREA^*^INTERVENTION [*F*_(4,32)_ = 14.4, *p* < 0.001] suggest that frequency of target areas was different between two interventions. According to *post-hoc* analyses, BoNT injections were used more for the upper extremity than for the lower extremity in both proximal and distal areas. As compared to BoNT injections, phenol neurolysis were used more frequent for lower proximal and distal areas, but less for the upper distal areas. Phenol injections were relatively evenly distributed to the upper proximal (26.7% ± 6.5%), lower proximal (32.7% ± 5.9%), and lower distal (39.2% ± 6.5%) areas. Phenol neurolysis to the upper distal area was rare (1.4% ± 1.4%).

### Combined Phenol and BoNT Injections

Among the 44 patients who received both phenol and BoNT, there were eight patients who received both phenol and BoNT injections during the first round of injections. In all eight encounters, phenol was only given to the lower extremity, while BoNT were injected to muscles in both upper and lower extremities. BoNT and phenol injections targeted different muscle groups (“alternative”) in six encounters and same muscle groups (“complementary”) in two encounters, including hamstrings and tibialis posterior muscles. The average amount of BoNT was 504.1 units, ranging from 200 to 800 units. The average phenol dose was 9.9 ml, with a range from 3 to 18 ml. The average interval from stroke onset to the procedure was 15.5 weeks, with five procedures within 3 months after stroke. Of note, one clinician performed injections for seven out of eight patients. A total of eight clinicians performed phenol neurolysis procedures.

### Side Effects

No side effects after phenol or BoNT injections in the early stage were reported in the chart review.

## Discussion

This retrospective chart review study is the first study on the early phenol neurolysis for spasticity management after stroke, to our knowledge. In this study, we summarized clinical practice patterns of early phenol neurolysis. The finding of decreased total dose of BoNT in the subsequent injection after early phenol neurolysis is of particular clinical interest.

### Characteristics of Early Use of Phenol Neurolysis in Management of Post-stroke Spasticity

For those who received phenol neurolysis within 15 months after stroke (*N* = 68), more than half (52.9%) received injections within 3 months, i.e., early injection. The average interval to receive first phenol injection was 16.3 weeks after stroke. The earliest date of phenol injection was 19 days (2.7 weeks) after stroke. The timeline was consistent with early use of BoNT injections. The mean time to receive BoNT injections was 18 days in a recent study (5). About one third of patients who received BoNT injections had first BoNT injections within 3 months (12). The dose of phenol in this study was 7.4 ml, with a range of 1–20 ml in a procedure. This was in general consistent with our earlier report of general practice pattern (21). In the previous study, patients with spasticity from all etiologies, mainly in the chronic stage, were included. The average phenol dose for a procedure was 10.95 ml. The range was 1–30 ml. Distributions of targeted nerves in this study were also similar to the previous report (21). Most frequently targeted nerves and nerve branches were tibial nerve and sciatic nerve, followed by obturator nerve, pectoral nerve and musculocutaneous nerve. In both this study and previous study (21), phenol neurolysis to the nerves and nerve branches of the upper extremity distal muscles was rarely performed. In another study, we reported more phenol cases (*N* = 57) for the upper extremity distal muscles (22). This is likely attributed to different etiologies. About half of the patients have a history of traumatic brain injury with severe spasticity in the hand and fingers in the previous study (22).

As reported in the literature (25, 26), we also reported combined use of BoNT and phenol in the procedure in this study. As previously reported (26), both phenol and BoNT injected targeted the same muscle groups, including knee flexors. In contrast to previous reports that phenol neurolysis was used in both upper and lower extremities, such as shoulder adductors, elbow flexors, hip adductors, and knee flexors (25, 26), only nerves in the lower extremity were targeted in this study. It is important to point out that the same clinician performed seven out of eight procedures with combined use of BoNT and phenol, while total eight clinicians performed other phenol procedures. This combined use is likely a reflection of personal preference.

### Phenol Neurolysis vs. BoNT Injections in the Early Stage

It is unique that we were able to compare the Phenol-First group and the BoNT-First group in this study. However, due to the nature of chart review study, it was not possible to assess the efficacy of phenol or BoNT injections with commonly used clinical scales, such as modified Ashworth scale. As used in our recent study, the amount of total BoNT dose is an alternative objective outcome assessment after the intervention (12).

It is of particular clinical interest that the total dose of BoNT was significantly decreased in the subsequent injection, if phenol neurolysis was performed first. The total phenol dose, however, was not significantly different between the Phenol-First group (5.9 ml) and the BoNT-First group (8.3 ml). The interval between the first injection and the subsequent injection was slightly longer, but not statistically significant, in the Phenol-First group (18.3 weeks) than in the BoNT-First group (10.7 weeks). These long intervals suggest that the subsequent injection (type and dose) was not planned during the first procedure. Therefore, it is plausible that phenol neurolysis was more effective in reducing spasticity than BoNT injections. Findings from our recent study support this explanation. In the subsequent injection, the total dose of BoNT was significantly increased after first BoNT injection in the early stage (12). Alternatively, the subsequent BoNT injections may be used to target areas that were not covered by phenol neurolysis in the Phenol-First group, such as the distal muscles in the upper extremity (Figure 3). As such, the total BoNT dose did not reflect the efficacy of phenol neurolysis in the Phenol-First group. Further studies are needed to support or dispute either explanation.

### Limitations

There are a few limitations in this retrospective chart review study. There was a lack of clinical assessment of spasticity, e.g., Ashworth Scale, Tardieu Scale, and we were not able to compare outcomes of phenol vs. BoNT injections. The total BoNT dose and the interval between first and second injections were able to provide some insights regarding the efficacy in spasticity reduction. The lack of assessment of clinical outcomes, especially muscle weakness and motor impairment and functional recovery, may cast some doubts into early phenol use. Both phenol neurolysis and BoNT injections cause muscle weakness, in addition to spasticity reduction. A recent study on early BoNT injections have shown that BoNT injections slows down development of spasticity, but without affecting motor recovery (5). It is generally accepted that phenol neurolysis has longer duration of spasticity reduction effect than BoNT injections (20). Whether weakness associated with long-lasting effect after early use of phenol neurolysis has adverse effects on motor recovery remains unknown. No documented side effects were found in this chart review study. This could be due to that fact that combined electrical stimulation and ultrasound imaging guidance were used in phenol neurolysis which allows a precise localization and reduced side effects (22). However, side effects from injections (phenol or BoNT) may be underestimated. Minor side effects may not be documented in the chart. Nevertheless, our findings on early phenol use help design further future studies.

## Conclusion

In summary, phenol neurolysis was started as early as 19 days after stroke. On average, patients received first phenol about 4 months after stroke with an average of 7.3 ml phenol. Early use of phenol neurolysis likely decreases the total amount of BoNT for management of post-stroke spasticity without increased side effects.

## Data Availability Statement

The original contributions presented in the study are included in the article/supplementary material, further inquiries can be directed to the corresponding author/s.

## Ethics Statement

The studies involving human participants were reviewed and approved by CPHS of University of Texas Health Science Center - Houston. Written informed consent for participation was not required for this study in accordance with the national legislation and the institutional requirements.

## Author Contributions

SL, JW, and MM contributed to conception, design of the study, and analyzed the data. JW organized the database. SL performed the statistical analysis and wrote the first draft of the manuscript. All authors contributed to manuscript revision, read, and approved the submitted version.

## Author Disclaimer

SL is consultant for SAOL therapeutics for research protocol development of a new phenol.

## Conflict of Interest

The authors declare that the research was conducted in the absence of any commercial or financial relationships that could be construed as a potential conflict of interest.

## Publisher's Note

All claims expressed in this article are solely those of the authors and do not necessarily represent those of their affiliated organizations, or those of the publisher, the editors and the reviewers. Any product that may be evaluated in this article, or claim that may be made by its manufacturer, is not guaranteed or endorsed by the publisher.
